# Turkish adaptation of the Nurse Team Resilience Scale in public health emergencies: a validity and reliability study

**DOI:** 10.1186/s12912-026-05004-0

**Published:** 2026-07-08

**Authors:** Dilek Yıldırım Gürkan, Rukiye Çakmak

**Affiliations:** 1 Department of Nursing, Faculty of Health Sciences, Yozgat Bozok University, Yozgat, Türkiye; 2 Medical Documentation and Secretarial Program, Department of Medical Services and Techniques, Vocational School of Health Services, Yozgat Bozok University, Yozgat, Türkiye

**Keywords:** Nurse team resilience, Public health emergencies, Scale adaptation, Validity, Reliability, Psychometric evaluation, Public health nursing

## Abstract

**Background:**

Public health emergencies place substantial pressure on healthcare systems and highlight the critical role of nursing teams in maintaining service continuity. Although resilience has frequently been examined at the individual level, instruments assessing resilience at the nursing team level remain limited. This study aimed to translate the Nurse Team Resilience Scale (NTRS) into Turkish and evaluate its psychometric properties among nurses working in primary healthcare settings.

**Methods:**

This methodological study was conducted with 218 nurses working in family health centers and community health centers between November 2025 and January 2026. The psychometric evaluation was guided by COSMIN methodological standards. The cross-cultural adaptation process included forward–backward translation, expert review, and pilot testing. Content validity was assessed using the content validity index. Construct validity was evaluated using exploratory factor analysis (EFA) and confirmatory factor analysis (CFA). Reliability was examined through internal consistency, split-half reliability, and test–retest analysis.

**Results:**

Content validity findings supported the cultural and conceptual appropriateness of the Turkish version, with I-CVI and S-CVI/Ave values of 1.00. EFA supported a single-factor structure explaining 72.685% of the total variance. CFA indicated acceptable to good model fit. The scale demonstrated high internal consistency, strong split-half reliability, and strong test–retest reliability, supporting short-term temporal stability.

**Conclusions:**

The Turkish version of the NTRS demonstrated strong psychometric evidence supporting its validity and reliability for assessing nursing team resilience in public health emergency contexts. The scale may be used in research and practice to evaluate and strengthen team resilience among nurses.

**Clinical trial number:**

Not applicable.

## Introduction

Public health emergencies (PHEs) encompass events that place sudden and intense pressure on health systems, including infectious disease outbreaks, natural disasters, and large-scale crises. The COVID-19 pandemic clearly demonstrated the central role of nurses in maintaining the sustainability of healthcare systems [1]. During the pandemic, high levels of depression, anxiety, and sleep disturbances were reported among healthcare workers, with nurses identified as being at particularly high psychosocial risk [2, 3]. Recent post-pandemic systematic reviews indicate that burnout, psychological distress, and workload pressures among nurses persist beyond the acute crisis period [4, 5].

Primary healthcare services constitute one of the key service areas where first contact with the community is established during public health emergencies, preventive health services are maintained, and local health needs can be monitored at an early stage [1]. Family health centers and community health centers occupy an important position in sustaining community-based healthcare services and monitoring at-risk groups during crisis periods [1]. In these settings, nurses’ capacity for coordination, mutual support, and collective action within teams is critical for the continuity of the response to public health emergencies. Protecting and supporting the nursing workforce constitutes a fundamental component of health systems’ capacity to respond effectively to crises. Ensuring that frontline healthcare workers, particularly nurses, are supported and that their psychological well-being is protected is considered critical for the sustainability of healthcare delivery [6]. Indeed, healthcare workers’ well-being has been shown to be directly associated with overall health system resilience [7]. In this context, resilience should be conceptualized not only as an individual psychological trait but also as a key workforce indicator reflecting a system’s preparedness and adaptive capacity in the face of crises [7].

Resilience is defined as the capacity of an individual to adapt to adverse conditions and recover following stress or disruption. It becomes observable particularly when individuals are exposed to significant challenges and operates at both individual and team levels [8]. Studies conducted among healthcare professionals have demonstrated that higher levels of resilience are associated with lower levels of burnout and psychological distress [9, 10]. These findings indicate that resilience is an important construct that should be assessed in terms of healthcare workers’ well-being and service continuity under crisis conditions [7, 9, 10]. However, existing adaptation studies in Türkiye have largely focused on individual resilience and psychological adjustment processes [11–13]. The team-based nature of healthcare delivery, particularly during crisis periods, makes it necessary to measure resilience not only at the individual level but also at the team level [8, 14]. Therefore, there is a need for context-specific, valid, and reliable measurement tools that can assess resilience among nursing teams [15].

The Nurse Team Resilience Scale (NTRS), developed by Su et al., is one of the measurement tools that responds to this need [15]. The scale focuses on assessing nursing teams’ capacity to adapt to challenges, maintain team functioning, and recover after adverse conditions in the context of public health emergencies [15]. Unlike tools that measure individual resilience, this scale considers the team-based nature of nursing services and conceptualizes resilience as a collective capacity [15]. Therefore, adapting the scale into Turkish may address an important gap in evaluating the resilience of nursing teams working in primary healthcare services against public health emergencies.

However, to our knowledge, no study has examined the cross-cultural adaptation and psychometric properties of the Turkish version of the scale. Since resilience measurement tools in Türkiye predominantly focus on the individual level, there remains an important gap in assessing the collective resilience of nursing teams [11–13, 15].

Accordingly, the aim of the present study is to adapt the Nurse Team Resilience Scale into Turkish and to evaluate its psychometric properties among nurses working in primary healthcare services in the context of public health emergencies.

## Methods

### Study design

This study was conducted as a methodological study to adapt the Nurse Team Resilience Scale into Turkish and to evaluate its psychometric properties. The study process included translation, cultural adaptation, content validity assessment, pilot testing, construct validity assessment, and reliability analyses. In the psychometric evaluation process, the Consensus-based Standards for the Selection of Health Measurement Instruments (COSMIN) criteria were considered as a methodological standards framework for systematically assessing the validity and reliability properties of measurement instruments [16].

### Participants

Data were collected between 15 November 2025 and 31 January 2026 from nurses working in Family Health Centers and Community Health Centers in the city center and districts of Yozgat, Türkiye. In scale validity and reliability studies, a sample size of at least 5–20 participants per item is recommended, while a minimum of 200 participants is suggested for confirmatory factor analysis (CFA) [17]. Accordingly, 218 nurses were included in the study.

In this study, a public health emergency was operationally defined as infectious disease outbreaks, pandemic conditions, natural disasters, or crisis situations that disrupt routine community-level healthcare delivery and require rapid organization and team-based response. Participants’ experience of a public health emergency was evaluated based on having worked during or professionally experienced the effects of such processes on healthcare delivery, particularly during community-level crises such as the COVID-19 pandemic.

Participants were included if they met the following criteria: (i) being a licensed nurse, (ii) working in a Family Health Center or Community Health Center, (iii) having worked during a process defined as a public health emergency or having professionally experienced its effects on healthcare delivery, and (iv) agreeing to participate in the study.

### Data collection tools

Data were collected through face-to-face interviews using the Nurse Descriptive Information Form and the Nurse Team Resilience Scale in the context of Public Health Emergencies.

### Nurse descriptive information form

This form, developed by the researchers, consisted of 11 items and included sociodemographic and professional characteristics such as age, gender, education level, years of professional experience, and working conditions.

### Nurse team resilience scale in the context of public health emergencies

The scale was developed by Su et al. in 2023 [15]. The original scale was developed to assess the collective resilience of nursing teams during public health emergencies and, unlike individual resilience measures, focuses on team members’ capacity to adapt together to challenges, maintain team functioning, and recover after adverse conditions [15]. In the original study, the scale development process was based on the theoretical framework of team resilience and a review of existing measurement tools [15]. It consists of eight items rated on a 5-point Likert scale ranging from 1 (“strongly disagree”) to 5 (“strongly agree”). There are no reverse-coded items, and the scale has a single-factor structure. The original study reported a Cronbach’s alpha of 0.94 [15]. Total scores range from 8 to 40, with higher scores indicating greater nursing team resilience. A total score above 24 indicates high team resilience [15].

### Validity analyses

Linguistic validity, cultural appropriateness, content validity, and construct validity of the scale were evaluated.

### Linguistic and cultural adaptation

In cross-cultural scale adaptation studies, it is recommended to evaluate not only word-level translation but also the semantic, conceptual, and contextual appropriateness of items in the target language [18–20]. Accordingly, the original English version of the scale was independently translated into Turkish by one linguist and two bilingual academics. The translations were compared by the research team, and a single Turkish version was developed by considering semantic equivalence, conceptual appropriateness, compatibility with the nursing context, and clarity of Turkish wording. This version was then back-translated into English by a different bilingual linguist who had not seen the original scale.

In addition to linguistic equivalence, cultural appropriateness was also evaluated. In this process, the items were examined in terms of their suitability for the Turkish nursing practice context, the team-based functioning of primary healthcare services, and the professional language used in public health emergency settings. Based on expert feedback, minor wording and clarity revisions were made without changing the meaning of the items, and the item content was preserved. Thus, both linguistic equivalence and conceptual appropriateness within the Turkish cultural and nursing context were supported.

### Content validity

The translated Turkish form was evaluated by nine experts with doctoral degrees in public health nursing and psychiatric nursing. Experts were asked to rate each item on a 4-point scale ranging from 1 = requires major revision to 4 = very appropriate. In the expert evaluation, linguistic accuracy, conceptual equivalence, cultural appropriateness, clarity, and the extent to which each item represented the construct of nursing team resilience were considered. Ratings of 3 or 4 were considered content-valid. The item-level content validity index (I-CVI) was calculated by dividing the number of experts rating each item as 3 or 4 by the total number of experts. The scale-level content validity index based on the average method (S-CVI/Ave) was calculated as the mean of the I-CVI values across all items. Values ≥ 0.80 were considered sufficient for expert agreement [18, 19, 21].

### Pilot testing

After content validity assessment, the scale was pilot tested with 10 nurses who had characteristics similar to the target sample. Participants reported that the items were clear and understandable, and no negative feedback was received. These nurses were not included in the main sample. The pilot testing was conducted to evaluate the clarity of Turkish wording, response feasibility, cultural and professional contextual appropriateness, and suitability of the administration time.

### Construct validity

Construct validity was evaluated using exploratory factor analysis (EFA) and confirmatory factor analysis (CFA). Sampling adequacy was examined using the Kaiser–Meyer–Olkin (KMO) test and Bartlett’s test of sphericity. A KMO value > 0.60 and a significant Bartlett’s test result at *p* < 0.05 were considered appropriate for factor analysis. Principal axis factoring with promax rotation was used in EFA. Factors with eigenvalues ≥ 1 and items with factor loadings ≥ 0.40 were accepted [18, 19, 21].

In CFA, model fit was evaluated using χ²/df, RMSEA, GFI, CFI, TLI, IFI, and SRMR fit indices. Acceptable fit criteria were considered as χ²/df < 5, RMSEA < 0.08, SRMR < 0.08, and other fit indices > 0.90 [18, 19, 22]. Since the data did not meet the assumption of multivariate normality, the Bootstrap Maximum Likelihood method was used in CFA. In addition, the Bollen–Stine bootstrap p value was examined for the bootstrap-based assessment of model fit.

### Reliability analyses

The reliability of the scale was evaluated using internal consistency, split-half reliability, and test–retest reliability. Internal consistency was examined using Cronbach’s alpha coefficient, and values ≥ 0.70 were considered acceptable [18–20, 23]. Split-half reliability was analyzed using the Spearman–Brown and Guttman split-half coefficients [18, 19, 21]. Test–retest reliability was assessed by re-administering the scale to 50 nurses two weeks after the initial application.

### Statistical analysis

Data were analyzed using IBM SPSS Statistics 24 and IBM AMOS 24. Descriptive statistics were presented as numbers, percentages, means, and standard deviations. Distributional characteristics of the data were evaluated using skewness and kurtosis values. Multicollinearity was examined before CFA, and no multicollinearity problem was detected. EFA was conducted using a correlation matrix, whereas CFA was conducted using a covariance matrix. Since the assumption of multivariate normality was not met, the Bootstrap Maximum Likelihood method was preferred in CFA, and the Bollen–Stine bootstrap p value was evaluated for reporting. Statistical significance was set at *p* < 0.05.

### Ethical considerations

Permission was obtained from the original developer for the adaptation of the scale from English to Turkish. Ethical approval was obtained from the Yozgat Bozok University Social and Human Sciences Ethics Committee (Approval No: E-39243114-200-353076). Institutional permission was also obtained from the Yozgat Provincial Health Directorate (Approval No: E-88517772-770-293937795). All procedures involving human participants were conducted in accordance with the relevant ethical guidelines and regulations and with the Declaration of Helsinki. Written informed consent was obtained from all participating nurses before the data collection process.

## Results

The sample consisted of 218 nurses. The mean age of the nurses was 31.96 years (SD = 6.85). More than half of the nurses (62.4%) were married. The mean years of professional experience was 9.01 years (SD = 6.87). Of the participants, 79.4% held a bachelor’s degree and 8.7% had postgraduate education.

### Content validity, cultural adaptation, and pilot testing

All eight items were rated as either 3 or 4 by all nine experts. Therefore, the I-CVI value was 1.00 for each item, and the S-CVI/Ave value was 1.00. The expert panel also confirmed that the items were semantically clear, conceptually equivalent, and culturally appropriate for the Turkish primary healthcare nursing context. Minor wording revisions were made to improve clarity and contextual suitability for family health centers and community health centers, without changing the conceptual meaning of the items. No item was removed from the scale. In the pilot testing with 10 nurses, no problems were reported regarding item clarity, comprehensibility, or administration feasibility.

The Kaiser–Meyer–Olkin (KMO) coefficient was 0.935, and Bartlett’s test of sphericity yielded χ² = 1514.612 (*p* < 0.001). Exploratory factor analysis indicated a single-factor structure. The scale explained 72.685% of the total variance. Factor loadings ranged from 0.762 to 0.921 (Table 1).

**Table 1:** Descriptive statistics and factor loadings of the eight items

	Mean	SD	Factor Loading
Item 1	3.64	1.25	0.83
Item 2	3.61	1.18	0.801
Item 3	3.61	1.15	0.762
Item 4	4.01	1.14	0.864
Item 5	3.88	1.19	0.921
Item 6	3.86	1.19	0.866
Item 7	3.96	1.23	0.904
Item 8	3.94	1.18	0.854

Total Variance Explained %72.685

The CFA results of the Nurse Team Resilience Scale in the context of public health emergencies are presented in Table 2. Multivariate normality was assessed and the data were found not to follow a normal distribution (critical value = 27.142). Therefore, the Bootstrap Maximum Likelihood estimation method was used.

**Fig. 1:**
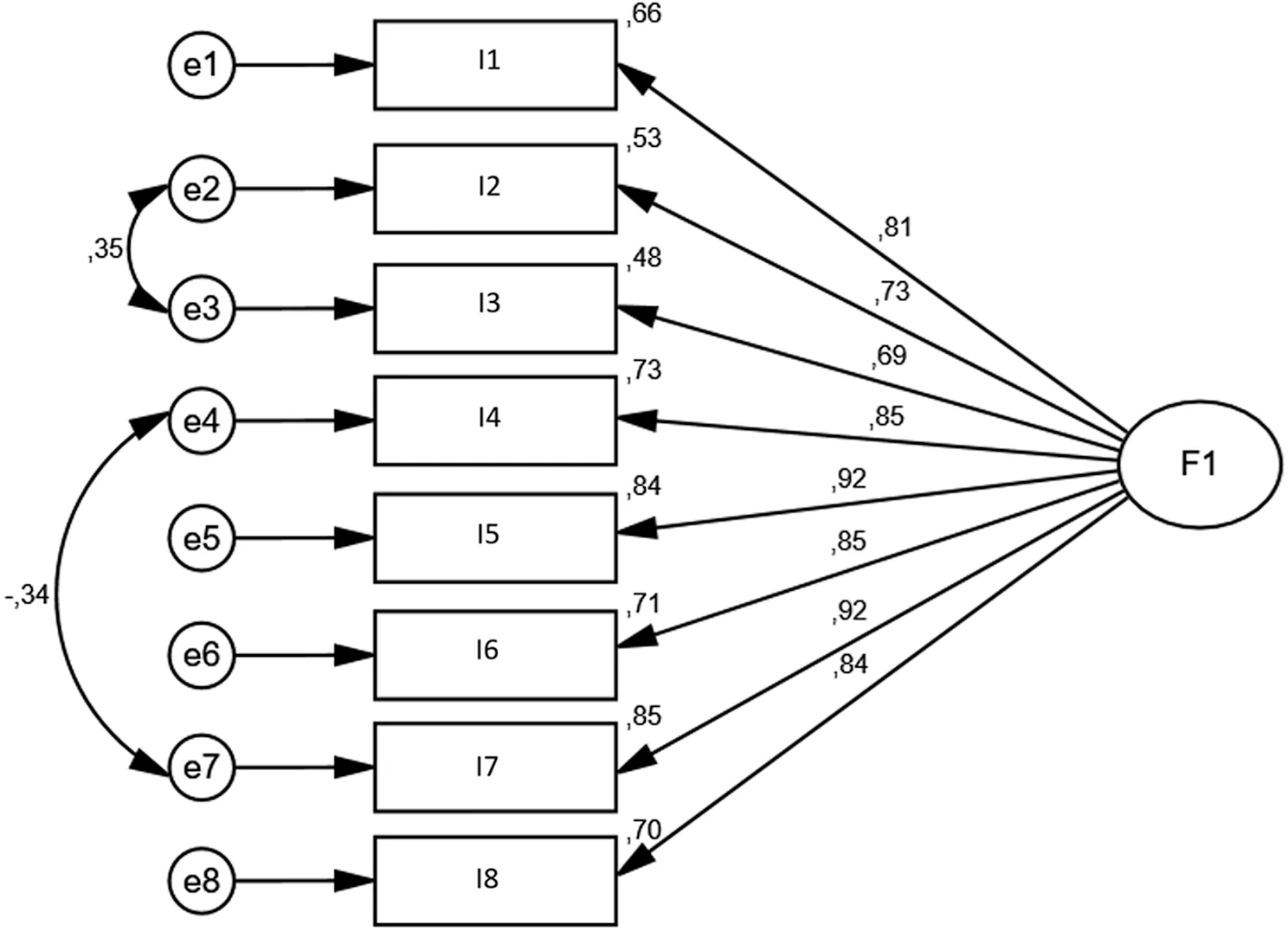
Standardized beta coefficients

Model fit indices indicated an acceptable to good fit for the single-factor model (CMIN/DF = 1.841, GFI = 0.964, IFI = 0.990, TLI = 0.984, CFI = 0.990, RMSEA = 0.062, SRMR = 0.020). In addition, the Bollen–Stine Bootstrap test supported the model fit (*p* = 0.386). These findings supported the single-factor structure of the Turkish version of the scale. All standardized factor loadings were statistically significant (*p* < 0.001). The standardized factor loadings are presented in Fig. 1.

**Table 2:** Confirmatory factor analysis results of the Nurse Team Resilience Scale in the context of public health emergencies

	β^1^	(95% CI)	S.E	*p*	*R* ^2^
I1	0.81	(0.74–0.86)	0.078	**< 0.001**	0.659
I2	0.73	(0.62–0.80)	0.074	**< 0.001**	0.534
I3	0.69	(0.57–0.78)	0.070	**< 0.001**	0.477
I4	0.85	(0.78–0.90)	0.065	**< 0.001**	0.727
I5	0.91	(0.88–0.94)	0.076	**< 0.001**	0.844
I6	0.84	(0.75–0.90)	0.070	**< 0.001**	0.715
I7	0.92	(0.88–0.94)	0.082	**< 0.001**	0.848
I8	0.83	(0.76–0.89)	---	**< 0.001**	0.701

¹Standardized beta coefficient; 95% CI: Bootstrap 95% Confidence Interval

### Reliability analysis

The Cronbach’s alpha coefficient for the entire scale was 0.946. According to the split-half reliability analysis, the Cronbach’s alpha value was 0.904 for the first half and 0.894 for the second half. The correlation between the two halves was 0.887. The Spearman–Brown correlation coefficient was 0.940, and the Guttman split-half coefficient was also calculated as 0.940 (Table 3).

**Table 3:** Reliability analyses of the scale

		Split-half analysis				
	Cronbach’s alpha	Cronbach’s alpha (first half)	Cronbach’s alpha (second half)	Spearman–Brown coefficient	Guttman split-half coefficient	Correlation between the two halves
Total scale	0.946	0.904	0.894	0.940	0.940	0.887

### Test–retest reliability

To evaluate the temporal stability of the Turkish version of the Nurse Team Resilience Scale (NTRS), the scale was re-administered to a subsample of 50 participants two weeks after the initial administration. The analysis revealed a statistically significant positive correlation between the test and retest scores (*r* = 0.96, *p* < 0.001). These findings support the short-term temporal stability of the Turkish version of the scale.

## Discussion

This study adapted the Nurse Team Resilience Scale into Turkish and evaluated its psychometric properties among nurses working in primary healthcare settings in the context of public health emergencies. The findings supported the single-factor structure, construct validity, internal consistency, split-half reliability, and short-term test–retest reliability of the Turkish version. These results indicate that the Turkish version of the Nurse Team Resilience Scale provides strong psychometric evidence supporting its validity and reliability for assessing nursing team resilience in primary healthcare settings during public health emergency contexts [15, 16, 24].

The single-factor structure identified in this study is consistent with the original NTRS development study [15]. The original scale was designed to assess nursing teams’ collective capacity to adapt to adversity, maintain team functioning, and recover after disruption in public health emergencies [15]. In the original study, the scale showed a single-factor structure and explained 72.33% of the total variance [15]. In the present study, the single-factor structure was also supported, and the scale explained 72.685% of the total variance. This similarity supports that the core conceptual structure of the scale was preserved in the Turkish nursing sample [15].

A recent Persian adaptation of the NTRS also shows that the scale has begun to be used across different cultures and high-stress nursing contexts [25]. In the Persian adaptation study, the scale was evaluated among Iranian nurses in the context of cardiopulmonary resuscitation, and a two-factor structure was identified [25]. In contrast, the present study supported a single-factor structure, consistent with the original NTRS development study [15, 25]. The emergence of a two-factor structure in the Persian study suggests that the factor structure of the NTRS may be sensitive to cultural context, clinical setting, and the nature of the crisis context [25]. Therefore, although the present findings support the structural validity of the Turkish version, further testing across different institutions, regions, and crisis contexts would be useful [16, 24, 25].

The exploratory factor analysis showed that item factor loadings ranged from 0.762 to 0.921, indicating that the eight items contributed strongly to the construct of nursing team resilience [18, 24]. The Kaiser–Meyer–Olkin value of 0.935 and the significant Bartlett’s test of sphericity supported the suitability of the data for factor analysis [18, 22]. Confirmatory factor analysis also indicated acceptable to good model fit for the single-factor model [22, 24]. Since the data did not meet the assumption of multivariate normality, the use of Bootstrap Maximum Likelihood estimation was an appropriate statistical choice [22, 24]. Reporting the Bollen–Stine Bootstrap p value in the Results section strengthened the transparency of the model fit assessment [22, 24]. Therefore, the CFA findings were interpreted together with both conventional fit indices and the Bollen–Stine Bootstrap result [16, 22, 24].

The reliability findings also supported the psychometric adequacy of the Turkish version. In this study, the Cronbach’s alpha coefficient was 0.946, which was very close to the value reported in the original NTRS study [15]. This result indicates that the items consistently measure the same construct [18, 23]. However, very high alpha values should be interpreted carefully because they may also reflect content similarity among items [18, 23]. The high split-half reliability coefficients further supported the internal consistency of the scale using another reliability approach [18, 21]. The high test–retest correlation obtained over a two-week interval supported the short-term temporal stability of the scale [16, 24]. This finding suggests that the scale may produce consistent scores over a short period when the underlying construct is not expected to change substantially [16, 24].

The cultural adaptation process is an important point in interpreting the findings of this study. Cross-cultural scale adaptation requires not only literal translation but also semantic, conceptual, and contextual equivalence between the original and target versions [18–20]. In this study, linguistic equivalence was supported through forward–backward translation procedures [18–20]. Cultural and professional appropriateness was examined through expert review and pilot testing [18–20]. The items were evaluated in terms of their suitability for the Turkish nursing practice context, the team-based functioning of primary healthcare services, and the professional language used in public health emergency settings. These procedures support the linguistic and conceptual appropriateness of the Turkish version for the target sample [18–20].

The findings should also be interpreted within the resilience literature in Türkiye. Existing resilience scales and adaptation studies in Türkiye have largely focused on individual resilience and psychological adjustment processes [11–13]. However, in Turkish social contexts, resilience may also be shaped through relational and collective processes rather than only as an individual psychological attribute [11, 26]. Recent qualitative evidence from Türkiye suggests that resilience may be associated with spirituality, cultural norms, personal meaning-making, and family support processes [26]. Although the present study focuses on professional nursing teams, this evidence supports the view that resilience may also operate through closely connected social units in Turkish contexts [26]. In this respect, the NTRS-TR may fill an important gap by assessing the collective resilience of nursing teams rather than individual resilience alone [15].

This cultural perspective strengthens the rationale for assessing resilience at the team level in nursing. Family health centers and community health centers are often characterized by small, interdependent teams that share responsibilities for preventive services, follow-up, health education, surveillance, and crisis response [1, 7]. In such settings, resilience is not only a matter of individual coping but also of coordination, mutual support, shared responsibility, and collective adaptation [7, 14, 15]. Team resilience has been conceptualized as a group’s capacity to maintain performance under pressure, adapt to changing conditions, and recover after adversity [14]. Therefore, assessing nursing team resilience may provide a culturally and professionally meaningful perspective beyond individual resilience in the Turkish primary healthcare context [14, 26].

Team resilience is increasingly considered an important construct for care continuity, patient safety, and service quality in healthcare [14, 27–29]. Resilient health systems require adaptive capacity not only at the individual level but also at team, organizational, and system levels during crises [7, 27, 29]. Teamwork processes such as communication, coordination, and leadership are closely associated with patient safety in healthcare settings [28]. Recent syntheses of healthcare resilience also emphasize that resilience should be understood as a multilevel process operating across individuals, teams, organizations, and systems [29]. Therefore, measuring nursing team resilience is consistent with contemporary approaches to health system resilience [7, 14, 27–29].

### Public health and managerial implications

The findings of this study have practical implications for nursing management and public health preparedness. During public health emergencies, brief and feasible measurement instruments are needed because of time constraints, workload pressure, and limited human resources [1, 7]. The eight-item structure of the NTRS-TR may allow rapid assessment of nursing team resilience in field settings [15]. Nurse managers and public health administrators may use the scale to identify teams that require additional organizational or psychosocial support [7, 15, 27]. Low team resilience scores should not be interpreted as individual failure but rather as indicators of team-level and organizational support needs [7, 27, 29].

When low team resilience is identified, the scale results can guide targeted team-based interventions. These interventions may include structured team debriefings after crisis-related events, clarification of team roles, simulation-based crisis response training, communication protocols, peer support mechanisms, and collective coping strategies [14, 30, 31]. Systematic review evidence indicates that healthcare team interventions can improve team effectiveness, especially when they include structured training, tools, organizational redesign, or multi-component programs [30]. Evidence-based teamwork frameworks such as TeamSTEPPS emphasize communication, team leadership, situation monitoring, and mutual support as teachable teamwork skills in healthcare settings [31]. These components are directly relevant to strengthening team resilience in primary healthcare teams during public health emergencies [14, 30, 31].

The Turkish version of the NTRS may also support future intervention and evaluation studies. For example, nurse managers may administer the scale before and after team-based training programs to evaluate whether collective coping, coordination, and mutual support improve over time [14, 15, 30]. The scale may also be used alongside measures of burnout, psychological distress, team cohesion, and individual resilience to better understand the relationship between team-level resilience and nurse well-being [9, 10, 14]. Previous studies have shown that resilience is associated with lower burnout and psychological distress among nurses and healthcare workers [9, 10]. Future research examining these relationships at both individual and team levels would strengthen the theoretical positioning of the NTRS-TR [14, 15, 29].

### Limitations

This study has several limitations. First, the study was conducted with nurses working in family health centers and community health centers in a single province, which may limit the generalizability of the findings to other regions and healthcare settings. Second, the use of face-to-face data collection may have increased the possibility of social desirability bias, particularly because the scale includes items related to team functioning. Third, convergent and discriminant validity were not evaluated in this study. Validation of measurement instruments is a multidimensional process that requires testing across different samples and in relation to theoretically associated constructs [16, 24]. Fourth, measurement invariance across groups such as gender, institution type, professional experience, or public health emergency exposure was not tested. Fifth, public health emergency experience was operationally defined based on professional exposure to processes such as pandemics, infectious disease outbreaks, disasters, or crisis conditions, but the intensity and type of exposure were not analyzed separately. Future studies should test the scale in larger and more diverse samples, compare different regions and institutional settings, examine convergent and discriminant validity, and evaluate measurement invariance across relevant subgroups [16, 24].

## Conclusion

In conclusion, the Turkish version of the Nurse Team Resilience Scale demonstrated strong psychometric evidence supporting its validity and reliability among nurses working in primary healthcare settings. The findings support the use of the scale for assessing nursing team resilience in the context of public health emergencies. By focusing on collective resilience rather than only individual resilience, the NTRS-TR may contribute to nursing management, public health preparedness, and future intervention studies aimed at strengthening team functioning during crises. Further research is needed to confirm its psychometric performance in different regions, healthcare settings, and professional groups.

## Data Availability

The datasets used and analyzed during the current study are available from the corresponding author upon reasonable request.

## Abbreviations

NTRS: Nurse Team Resilience Scale
EFA: Exploratory Factor Analysis
CFA: Confirmatory Factor Analysis
KMO: Kaiser-Meyer-Olkin Index
PHEs: Public health emergencies
CI: Confidence Interval
